# Evaluation of self-assembled HCPT-loaded PEG-*b*-PLA nanoparticles by comparing with HCPT-loaded PLA nanoparticles

**DOI:** 10.1186/1556-276X-9-687

**Published:** 2014-12-19

**Authors:** Xiangrui Yang, Shichao Wu, Yange Wang, Yang Li, Di Chang, Yin Luo, Shefang Ye, Zhenqing Hou

**Affiliations:** Department of Biomaterials, College of Materials, Xiamen University, Xiamen, 361005 China; Department of Chemistry, College of Chemistry & Chemical Engineering, Xiamen University, Xiamen, 361005 China

**Keywords:** 10-Hydroxycamptothecin, Self-assembly, PEG-*b*-PLA, Nanoparticles

## Abstract

We present a dialysis technique to prepare the 10-hydroxycamptothecin (HCPT)-loaded nanoparticles (NPs) using methoxypolyethylene glycol-poly(d,l-lactide) (PEG-*b*-PLA) and PLA, respectively. Both HCPT-loaded PEG-*b*-PLA NPs and HCPT-loaded PLA NPs were characterized by differential scanning calorimetry (DSC), dynamic light scattering (DLS), transmission electron microscopy (TEM), scanning electron microscopy (SEM) and confocal laser scanning microscopy (CLSM). The results showed that the HCPT-loaded PEG-*b*-PLA NPs and HCPT-loaded PLA NPs presented a hydrodynamic particle size of 120.1 and 226.8 nm, with a polydispersity index of 0.057 and 0.207, a zeta potential of −31.2 and −45.7 mV, drug encapsulation efficiency of 44.52% and 44.94%, and drug-loaded content of 7.42% and 7.49%, respectively. The HCPT-loaded PEG-*b*-PLA NPs presented faster drug release rate compared to the HCPT-loaded PLA NPs. The HCPT-loaded PEG-*b*-PLA NPs presented higher cytotoxicity than the HCPT-loaded PLA NPs. These results suggested that the HCPT-loaded PEG-*b*-PLA NPs presented better characteristics for drug delivery compared to HCPT-loaded PLA NPs.

## Background

Nowadays, cancer is one of the most common causes of death all over the world. Chemotherapy is still a commonly used strategy for cancer treatment [1, 2], but their efficacy is largely limited by low stability, short circulating half-life, and the toxicity associated with the anticancer drugs to normal tissues [3–6]. The nanoparticles (NPs) have been proposed to overcome these inconveniencies, for NPs can encapsulate a series of poorly water-soluble anticancer drugs and reduce their toxicity by the way of releasing them in a sustained manner at their target site [1, 7–10]. In addition, the self-assembly technique is a simple and low-cost method for producing NPs in a controllable way [11, 12]. Hence, the self-assembled NPs have attracted considerable interest for their potential use in anticancer drug delivery system.

Although the NPs enhanced the stability and decreased the toxicity of drugs, they only solved part of the problems, since the particles could be still rapidly cleared by the reticuloendothelial system (RES) uptake [11, 13]. It was pointed that hydrophobic NPs were more prone to RES uptake as compared to the negatively charged NPs [14]. So the NPs with surface modification have been devised to further improve the suboptimal pharmacokinetic properties [15–17]. Among all the strategies, PEGylation was proved to be the most effective, because of its unique physicochemical characteristics, such as good dispersibility and solubility [18–20]. With PEG chains on their surface, the NPs can entangle water molecules in the aqueous phase, which will render their surface hydrophilic and hinder protein binding and RES uptake [3]. It can still enhance their solubility and diminish their aggregation and immunogenicity [21]. More importantly, polyethylene glycol has been approved by FDA for human use. All these properties contribute to wide-spread use of PEG in many therapeutics [3, 16, 21].

10-Hydroxycamptothecin (HCPT) is a promising broad-spectrum antitumor agent which has shown to have a strong anti-tumor activity against gastric carcinoma, hepatoma, leukemia, and tumor of the head and neck in clinical practice [22]. In spite of its remarkable success in early clinical trials, the application of HCPT is still largely limited because of its poor solubility and stability, which led to low therapeutic efficiency and a number of side effects owing to the conversion of HCPT from active lactone form to the inactive carboxylate form under physiologic conditions [5, 23]. For these reasons, we chose HCPT as the model chemotherapeutic agent, expecting to further improve the suboptimal pharmacokinetic properties and promote its clinical application.

Moreover, testing tools are also important for future clinic use, and fluorescence imaging technique is an effective research tool to investigate many *in vivo* processes in the life sciences, such as the locations and sizes of tumors and the distribution of chemotherapy agents [24, 25]. The intrinsically fluorescence properties of HCPT in the drug delivery system will provide a good signal readout for the detection, which will meet the requirements of cancer diagnosis and therapy simultaneously.

In this paper, we presented a dialysis technique to direct the self-assembly of the HCPT-loaded PEG-*b*-PLA NPs. The hydrophobic polymeric core of the platform readily encapsulated the water-insoluble drug for systemic drug delivery. The physicochemical properties of the HCPT-loaded PEG-*b*-PLA NPs were characterized by differential scanning calorimetry (DSC), dynamic light scattering (DLS), scanning electron microscope (SEM), transmission electron microscopy (TEM), and confocal laser scanning microscopy (CLSM). *In vitro* drug release profiles and cytotoxicity tests were also conducted. The HCPT-loaded PLA NPs were also prepared and characterized in the same way and used for comparison.

## Methods

### Materials

All chemicals were analytical grade and used as received without further purification. The ultrapure water (18 MΩ∙cm^−1^) was used throughout the work. The 10-HCPT (purity >99%) was purchased from Lishizhen Pharmaceutical Co, Ltd (Wuhan, Hubei, China). PLA (50 kDa) and PEG-*b*-PLA (10%) were provided by Daigang BIO Engineer Co., Ltd. (Shandong, China). A dialysis bag (Mw cutoff = 8,000 to 14,000 Da) was ordered from Greenbird Inc. (Shanghai, China).

### Preparation of the HCPT-loaded PEG-*b*-PLA NPs and HCPT-loaded PLA NPs

The HCPT-loaded PEG-*b*-PLA NPs were prepared by a facile dialysis method. In brief, 100 mg of PEG-*b*-PLA was dissolved in 10 mL of acetone (solution A), and 10 mg of HCPT was dissolved in 0.5 mL of 0.01 M NaOH solution (solution B). Then solution B was dropped into solution A, and the mixture was used as the organic phase. Subsequently, the resulting organic phase was then introduced into a dialysis bag and dialyzed against 1,000 mL of water as the aqueous phase for 8 h. The as-prepared HCPT-loaded PEG-*b*-PLA NPs were lyophilized for 24 h using a freeze drier (Labconco Plus 12, Labconco, Kansas City, MO, USA) and stored at 4°C for use. The HCPT-loaded PLA NPs were prepared in a similar way using 100 mg of PLA. The drug-loaded content and drug encapsulation efficiency of both HCPT-loaded PEG-*b*-PLA NPs and HCPT-loaded PLA NPs were determined with ultraviolet spectrophotometry at 383 nm. The drug-loaded content and drug encapsulation efficiency were presented by the following equations:


### DSC analysis

The DSC analysis was performed on the solid samples using Netzsch model DSC-204 (Netzsch, Selb, Germany) with heating cycles of 0°C to 200°C. Samples (4 to 5 mg) were placed in a sealed aluminum pan and heated continuously at the rate of 10°C min^−1^ under a constant flow (40 mL min^−1^) of N_2_.

### Particle size, polydispersity index, surface charge, morphology, and HCPT distribution

The average particle size, zeta potential, and polydispersity index (PDI) were determined DLS using Malvern Zetasizer Nano-ZS (Malvern Instruments, Worcestershire, UK). The morphology of the HCPT-loaded PEG-*b*-PLA NPs was examined by SEM (LEO 1530; ZWL, Pegnitz, Germany) and TEM (JEM-2100; JEOL Ltd., Tokyo, Japan) at 20 and 200 kV, respectively. One drop of the suspension was placed on a silicon wafer or a carbon-coated copper grid and dried in the air before observation. The distribution of HCPT within the HCPT-loaded PEG-*b*-PLA NPs was analyzed by CLSM (Olympus FV 1000; Olympus, Tokyo, Japan). The HCPT-loaded PLA NPs were used for comparison.

### *In vitro*drug release behavior

The *in vitro* drug release studies of the HCPT-loaded PEG-*b*-PLA NPs were performed using the dialysis technique. The particles were dispersed in PBS (10 mL) and placed into a pre-swelled dialysis bag (MWCO 3,500 Da). The dialysis bag was then immersed in 0.1 M PBS at pH 7.4 and oscillated continuously in a shaker incubator (100 rpm) at 37°C. All samples were assayed by fluorescence spectrophotometry (HORIBA Fluoromax-4; HORIBA Ltd, Minami-ku, Kyoto, Japan). The HCPT-loaded PLA NPs were used for comparison.

### *In vitro*cell viability assays

Human hepatocellular carcinoma cells (BEL-7402) were cultured in standard cell media recommended by the American Type Culture Collection. The cells seeded in 96-well plates were incubated with a series of increasing concentrations of the HCPT-loaded PEG-*b*-PLA NPs for 48 h. Subsequently, the relative cell viability was assessed by the standard MTT assay. Cells treated with free HCPT and cells treated with the HCPT-loaded PLA NPs were compared.

## Results and discussion

### Preparation of the HCPT-loaded PEG-*b*-PLA NPs

**Figure 1:**
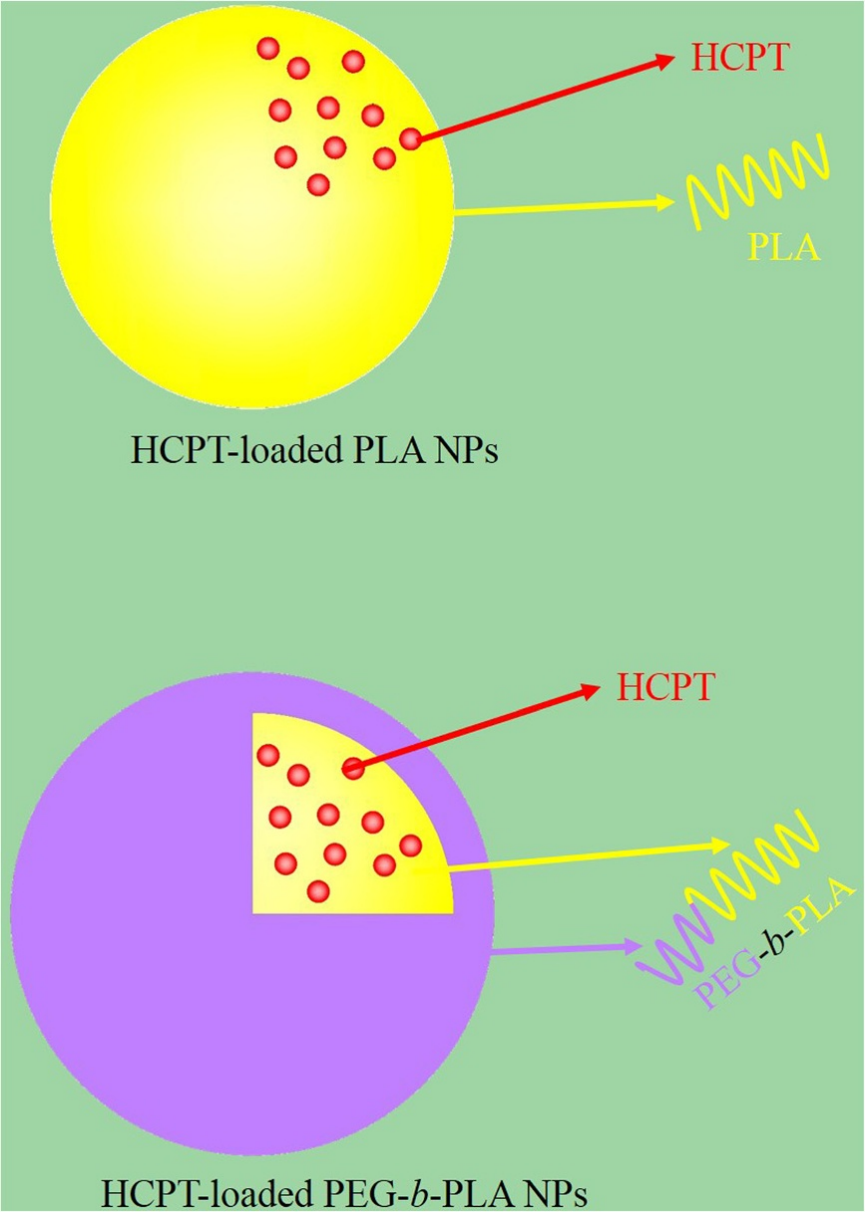
**Schematic representations of HCPT-loaded PLA NPs and HCPT-loaded PEG-** ***b*** **-PLA NPs.**

HCPT has a rather poor solubility for most organic solvents, so NaOH solution is used to dissolve HCPT. Acetone is water-miscible and a good solvent for PEG-*b*-PLA, so it is chosen as the organic phase. Firstly, PEG-*b*-PLA was dissolved in acetone (solution A) and HCPT was dissolved in NaOH solution (solution B). When solution B was dropped into solution A, PEG-*b*-PLA and HCPT were codissolved in this mixed solution, which was used as the organic phase and then extensively dialyzed against the aqueous phase. In the dialysis process, acetone was gradually removed and slowly replaced with water. To attain the minimal energy state, the hydrophobic PLA assembled together and formed a core while the hydrophilic PEG extended to the aqueous environment to form a shell (Figure 1). Because of the driving force of hydrophobic interaction, HCPT spontaneously transferred into the hydrophobic cores of the NPs. Although both of PEG-PLA and PLA particles were prepared by the same dialysis method, their formation mechanisms are different: the amphiphilic PEG-PLA formed micelles-like particles with core-shell structure; however, the homopolymer of PLA formed homogeneous solid particles. The determined drug entrapment efficiency and drug-loaded content of HCPT-loaded PEG-*b*-PLA NPs determined by ultraviolet spectrophotometry were 44.52% ± 0.42% and 7.42% ± 0.07%, respectively, and those of HCPT-loaded PLA NPs were 44.94% ± 0.54% and 7.49% ± 0.09%, respectively.

### DSC analysis

**Figure 2:**
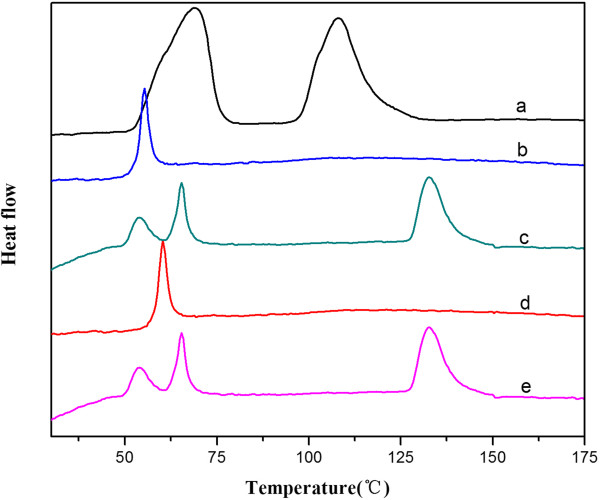
**The DSC patterns.** (a) Bulk HCPT. (b) PLA. (c) HCPT-loaded PLA NPs. (d) PEG-*b*-PLA. (e) HCPT-loaded PEG-*b*-PLA NPs.

The state of the incorporated drug in the HPLC-PEG-*b*-PLA NPs was proved using DSC (Figure 2). Under the experimental conditions, the DSC thermogram of the free HCPT had two endothermic peaks at 68.286 and 107.456°C, whereas that of the blank PEG-*b*-PLA particles showed a sharp endothermic peak at 53.253°C. In the curve of the HCPT-loaded PEG-*b*-PLA NPs, the peaks of HCPT and the peak of PEG-*b*-PLA were still existed, evidencing the presence of the crystalline drug in the HCPT-loaded PEG-*b*-PLA NPs. And the curves of PLA and HCPT-loaded PLA NPs also indicated the crystalline state of the incorporated drug in the HCPT-loaded PLA NPs.

### Particle size, PDI, surface charge, and morphology

**Figure 3:**
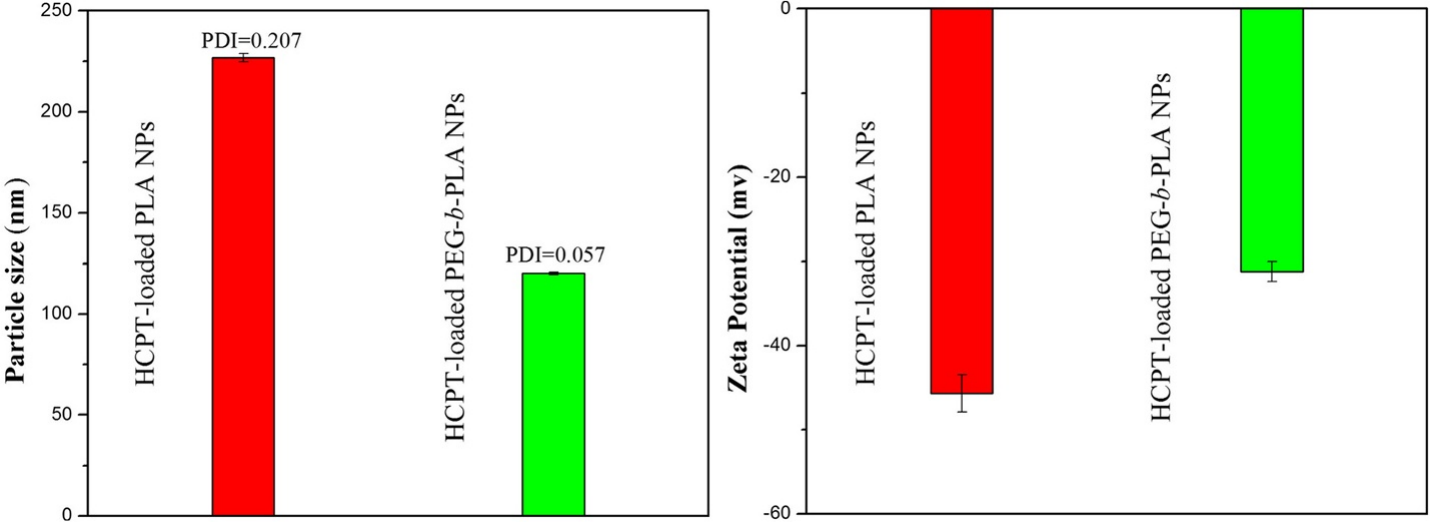
**Particle size and zeta potential.** Particle size and zeta potential of HCPT-loaded PEG-*b*-PLA NPs and HCPT-loaded PLA NPs.

Figure 3 showed that the particle size of the HCPT-loaded PEG-*b*-PLA NPs was 120.1 ± 4.9 nm, much smaller than that of the HCPT-loaded PLA NPs (226.8 ± 10.4 nm), indicating that the HCPT-loaded PEG-*b*-PLA NPs will be taken up easier by cancer cells, for smaller particle size favors EPR targeting (enhanced permeability and retention effect). The reason may be that the hydrophilicity of the polymer becomes stronger with PEGylation, which can attain lower energy state than that without PEGylation and form smaller particles. Moreover, due to its lower PDI (0.057), the HCPT-loaded PEG-*b*-PLA NPs would have a better dispersibility and stability than the HCPT-loaded PLA NPs. Figure 3 also showed that the HCPT-loaded PEG-*b*-PLA NPs and HCPT-loaded PLA NPs presented a zeta potential of −31.2 and −45.7 mV, for the oxygen atom of the PEG chains could combine with a few hydrogen ions, which was positively charged, and made the zeta potential lower. However, the zeta potential of the two NPs was high enough to support that these NPs could not aggregate much in aqueous state in general and in physiologically relevant media in particular.

**Figure 4:**
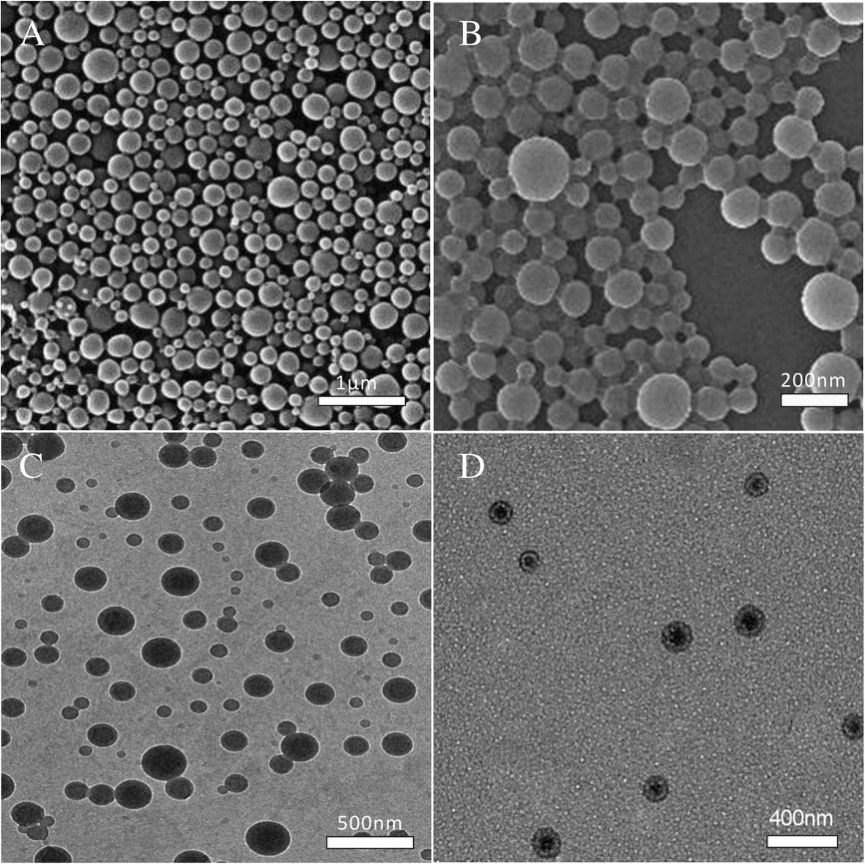
**Electron microscopy images.** SEM images of HCPT-loaded PLA NPs **(A)** and HCPT-loaded PEG-*b*-PLA NPs **(B)** and TEM images of HCPT-loaded PLA NPs **(C)** and HCPT-loaded PEG-*b*-PLA NPs **(D)**.

The SEM images revealed that the HCPT-loaded PEG-*b*-PLA NPs were regularly spherical in shape and had a relatively smooth surface with a little more than 100 nm in particle size, which was in accordance with the result of DLS. While the HCPT-loaded PLA NPs showed uniform color in TEM images, the HCPT-loaded PEG-*b*-PLA NPs contained a core particle with a lighter outer region (Figure 4), which might represent the hydrophobic core, made up of PLA and HCPT, and the hydrophilic shell, made up of PEG chain, respectively.

**Figure 5:**
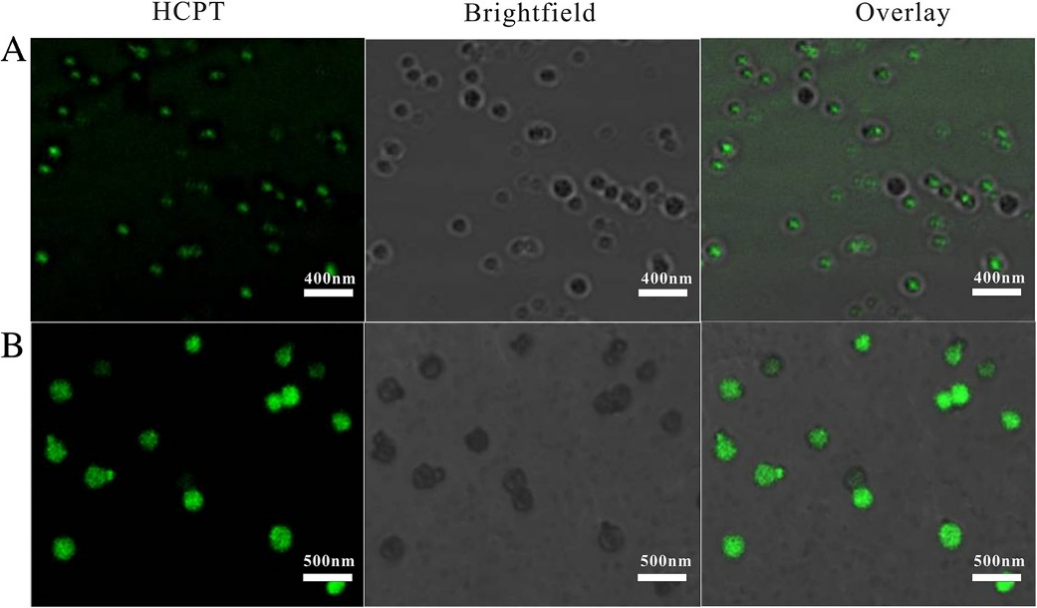
**CLSM images of HCPT-loaded PEG-** ***b*** **-PLA NPs (A) and HCPT-loaded PLA NPs (B).**

The distribution of HCPT was showed in Figure 5. The green fluorescence imaging (excitation at 382 nm) was performed to visualize HCPT in the NPs. In the HCPT-loaded PLA NPs, green fluorescence imaging overlapped nicely with the image of the particles, which indicated that HCPT was uniformly distributed within the particles. However, in the HCPT-loaded PEG-*b*-PLA NPs, the green fluorescence only appeared in the core of the NPs, demonstrating that HCPT only existed in the hydrophobic core of the particles. The results of the TEM and CLSM images both confirm the core-shell architectures of the HCPT-loaded PEG-*b*-PLA NPs: HCPT and PLA formed the core, and PEG formed the shell. With the hydrophilic shell, the steric stabilization of the NPs was enhanced in the dispersion, and the particle size was decreased.

### *In vitro*drug release behavior

**Figure 6:**
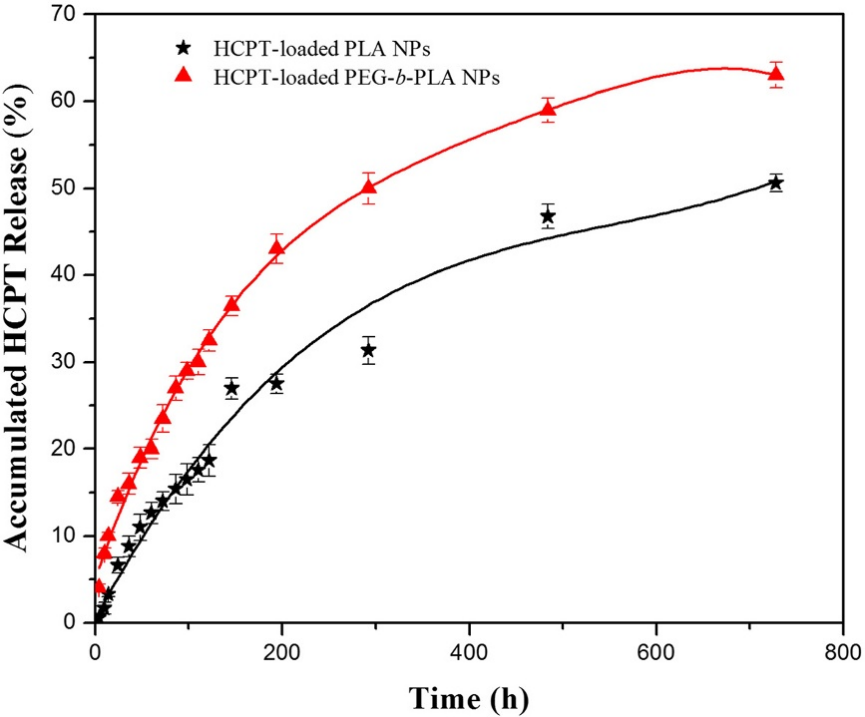
***In vitro*** **release profiles of HCPT-loaded PEG-** ***b*** **-PLA NPs and HCPT-loaded PLA NPs in PBS (1/15 M, pH 7.4).**

The release behavior of the NPs is an important aspect because this information is directly related to the design of the nanodrug delivery system. An *in vitro* release study of the HCPT-loaded PEG-*b*-PLA NPs (7.0% content) was performed and that of HCPT-loaded PLA NPs (6.9% content) was used as comparison. As shown in Figure 6, both two NPs exhibited a slow, prolonged release profile over a period of 750 h. While the release rate was a little faster, the total HCPT released from the HCPT-loaded PEG-*b*-PLA NPs was 63.0%, much higher than that of the HCPT-loaded PLA NPs. The profiles may be explained by two factors. Firstly, the particle size of the HCPT-loaded PEG-*b*-PLA NPs was much smaller than that of the HCPT-loaded PLA NPs, reducing the total releasing time of the drug from the NPs. Second, the hydrophilic PEG on the surface of the HCPT-loaded PEG-*b*-PLA NPs was used as a buffer role, which could reduce the hydrophobic interaction between the drug and polymer matrix and facilitate the release of the drug from the NPs core. Both of the factors could promote the release of HCPT from the HCPT-loaded PEG-*b*-PLA NPs.

### *In vitro*cell viability assays

**Figure 7:**
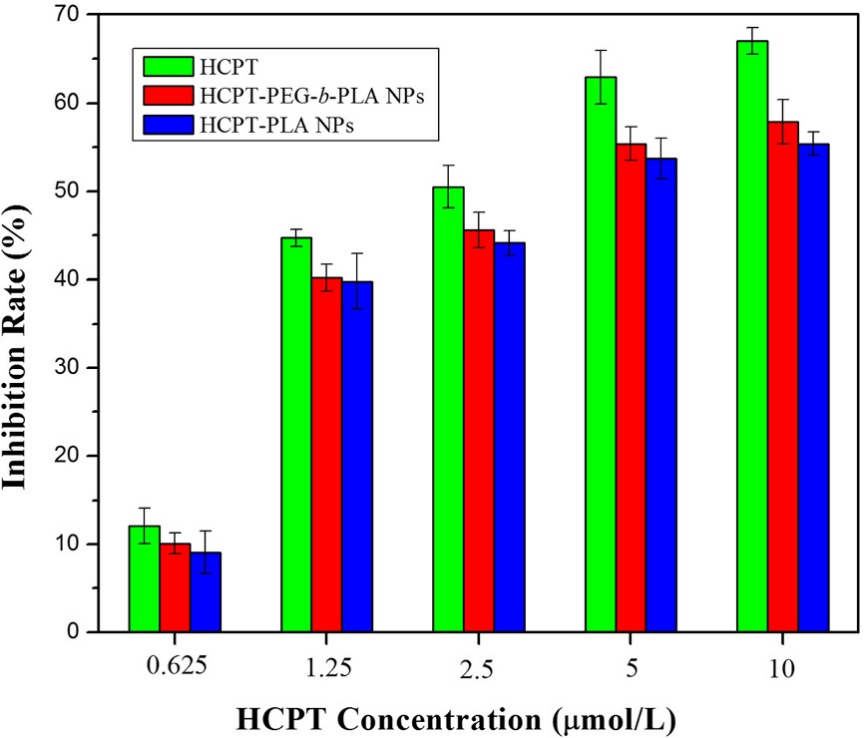
**The** ***in vitro*** **cytotoxicity assay against human liver BEL-7402 cells (48 h);** ***p*** **< 0.05.**

The cytotoxicity of the HCPT-loaded PEG-*b*-PLA NPs and HCPT-loaded PLA NPs was investigated and compared with bulk HCPT using BEL-7402 cells. As shown in Figure 7, at equivalent concentrations of HCPT, the HCPT-loaded PEG-*b*-PLA NPs showed higher cytotoxicity than the HCPT-loaded PLA NPs at equivalent concentrations of HCPT. The possible reasons are as follows. Firstly, the release rate of the HCPT-loaded PEG-*b*-PLA NPs was faster, and the total HCPT released was much more than HCPT-loaded PLA NPs (see Figure 6). Secondly, the particle size of the HCPT-loaded PEG-*b*-PLA NPs was much smaller, leading to the easier cellular uptake and more drug accumulation inside the cell and thus to the enhanced cytotoxicity. HCPT showed the highest inhibition rate, mainly ascribing to the fact that HCPT would directly act on the target site inside the cells without drug release with increasing intracellular drug concentration to a high level within 24 h.

## Conclusions

In this study, we developed a simple but successful method to obtain both HCPT-loaded PEG-*b*-PLA NPs and HCPT-loaded PLA NPs with fine characteristics for drug delivery. Although both exhibiting a slow, prolonged release profile, the HCPT-loaded PEG-*b*-PLA NPs presented a smaller particle size, faster drug release, and higher cell cytotoxicity compared to the HCPT-loaded PLA NPs. The animal experiments are going on now. The results obtained in this study indicate that these NPs might become a promising drug delivery system for HCPT.
